# Whole-Genome Sequencing and Comparative Genomic Analysis of *Citrobacter farmeri* and *Enterobacter cloacae* from Unhatched Green Turtle Eggs

**DOI:** 10.3390/vetsci13050462

**Published:** 2026-05-10

**Authors:** Nurcan Önen, Bahadır Törün, Can Yılmaz

**Affiliations:** 1 Biology Department, Institute of Graduate Studies, Hakkari University, Hakkari 30000, Turkey; onennurcan12@gmail.com; 2 Medical Services and Techniques Programme, Vocational School of Health Sciences, Hakkari University, Hakkari 30000, Turkey; canyilmaz@hakkari.edu.tr

**Keywords:** *Chelonia mydas*, WGS, *Citrobacter*, *Enterobacter*, one health

## Abstract

Bacteria associated with unhatched sea turtle eggs remain poorly characterized at the genomic level. This study provides genome-scale characterization of bacterial isolates recovered from unhatched green sea turtle (*Chelonia mydas*) eggs at Akyatan Beach—a critical nesting site in the Eastern Mediterranean. Sampling 30 nests during the nesting season, we isolated bacteria from infected eggshells and dead embryos. Following Matrix-Assisted Laser Desorption/Ionization Time-of-Flight Mass Spectrometry(MALDI-TOF MS) identification and 16S rRNA validation, we performed whole-genome sequencing (WGS) on *Citrobacter farmeri* and *Enterobacter cloacae*, two opportunistic pathogens of significant clinical and ecological concern. High-quality draft genomes revealed remarkable metabolic versatility, particularly within carbon and nitrogen pathways. Most notably, we identified extensive resistomes including resistance to β-lactams, fluoroquinolones, and aminoglycosides, alongside virulence factors for adhesion and iron acquisition. ANI analysis confirmed high genomic similarity to clinical reference strains, comparative genomic analysis revealed a substantial accessory gene pool, suggesting potential genomic flexibility between the two isolates. These findings provide the first genome-scale insight into these pathogens in *C. mydas* nests, and highlighting their genomic potential for opportunistic pathogenicity. Our results advocate for integrating genomic microbial surveillance into nesting beach management through a ‘One Health’ lens.

## Simple Summary

Sea turtle eggs that fail to hatch represent a significant conservation concern, yet the bacteria associated with these eggs remain poorly understood at the genomic level. In this study, we collected unhatched green sea turtle (*Chelonia mydas*) eggs from Akyatan Beach in Turkey—one of the most important nesting sites for this species in the Mediterranean—and isolated bacteria from infected eggshells and dead embryos. Two isolates, *Citrobacter farmeri* and *Enterobacter cloacae*, were selected for whole-genome sequencing based on their known clinical relevance. Genomic analysis revealed that both isolates carry gene sequences associated with resistance to multiple antibiotic classes and with virulence-related functions, though these findings represent genomic predictions that require phenotypic confirmation. Comparison with clinical reference strains showed high genomic similarity, highlighting the potential relevance of these bacteria within a One Health context. These findings provide a preliminary genomic baseline for bacteria recovered from unhatched sea turtle eggs and support the value of incorporating microbial monitoring into nesting beach conservation strategies.

## 1. Introduction

Marine ecosystems harbor an extraordinary diversity of life forms that have evolved over millions of years, playing indispensable roles in maintaining ecological balance. Among these, sea turtles represent an ancient lineage of marine reptiles that have persisted for over 200 million years with remarkable morphological stability [1,2]. Belonging to the order Testudines, sea turtles are primarily classified under two extant families: Cheloniidae and Dermochelyidae [3]. Of the seven currently recognized species, only *Caretta caretta* and *Chelonia mydas* regularly nest along the Mediterranean coasts of Turkey [4,5].

The conservation of migratory species inherently demands transboundary cooperation, as regional-scale threats can perturb critical life-history stages across disparate populations. To address this complexity, Regional Management Units (RMUs) were developed as a spatially explicit framework for evaluating the conservation status and anthropogenic pressures facing sea turtle nesting sites. Wallace et al. (2023) identified 48 established RMUs spanning six of the seven extant sea turtle species—excluding the flatback turtle—with 11 units specifically delineated for green turtles (*Chelonia mydas*) [6]. Focusing on the Mediterranean basin, recent evidence provided by Karaman et al. (2022) posits that a minimum of three distinct management units are essential for the effective oversight of *C. mydas* [7]. Despite localized challenges, green turtle populations have exhibited robust growth trajectories both on a global scale [8] and within the Mediterranean region [9,10]. For many years *C. mydas* has been classified as “endangered” by the International Union for Conservation of Nature (IUCN), primarily due to habitat degradation, climate change, illegal harvesting, pollution, and incidental bycatch [11]. However, in global *C. mydas* was updated to the “least concern” category by the IUCN in 2025, while Mediterranean subpopulations became “near threatened” [12].

Climate change has also emerged as a critical driver of population instability. Rising temperatures influence incubation conditions and hatchling sex ratios, with temperatures above 29 °C skewing hatchlings toward females [13,14]. Sea-level rise and coastal erosion threaten to diminish or eliminate viable nesting beaches [15,16]. These changes, when compounded with human-induced stressors, amplify the vulnerability of eggs and hatchlings to microbial colonization and disease.

In recent years, increasing attention has been paid to infectious diseases affecting sea turtle health. *C. mydas* is particularly susceptible to bacterial pathogens such as *Vibrio* spp., *Aeromonas* spp., and *Mycoplasma* spp., which are known to cause septicemia, ulcerative dermatitis, respiratory infections, and embryonic mortality [17,18,19]. These pathogens can infect turtles through direct contact with contaminated water, nesting sand, or via vertical transmission from infected females to eggs [20,21].

Environmental conditions at nesting sites significantly influence pathogen prevalence and virulence. For instance, high humidity and organic content in nesting sand can facilitate bacterial growth and penetration of the eggshell, leading to developmental arrest or embryo death [22]. Pathogens like *Salmonella* spp. and *Escherichia coli* have also been detected in nesting environments, raising concerns about zoonotic transmission and food safety, especially where turtle products are consumed [23,24]. Such infections may also compromise hatchling immunity, reducing survival rates post-emergence [25].

Despite growing interest in the role of bacteria in sea turtle health, much of the existing literature has focused on fungal agents such as *Fusarium* spp. and *Aspergillus* spp. [26,27]. In contrast, bacterial diversity—particularly in eggs—remains poorly characterized in many regions, including the Eastern Mediterranean. Addressing this knowledge gap is important, as bacteria with pathogenic potential have been associated with reduced hatching success and weakened early life stages in other sea turtle populations [28,29].

This study aims to identify and characterize the bacterial agents present in unhatched (failed) *C. mydas* eggs collected from one of the major nesting beaches in the Mediterranean region of Turkey. Notably, Akyatan serves as the largest rookery for the Mediterranean green turtle, supporting the nesting of an estimated 20% of this subpopulation [30]. By characterizing the bacteria associated with unhatched eggs, this research contributes to the development of targeted conservation and monitoring strategies. A better understanding of the bacterial diversity and genomic traits present in nesting environments is valuable for informing hatchery management.

## 2. Materials and Methods

### 2.1. Sample Collection

**Figure 1:**
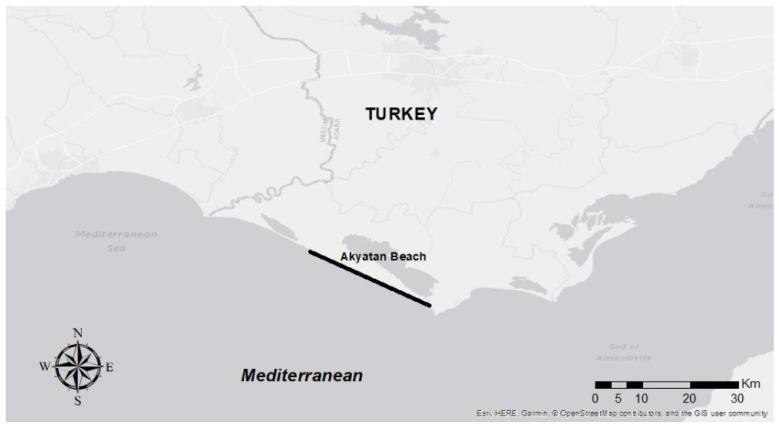
Sampling site on Akyatan Beach.

Samples in this study were collected from the green turtle nesting areas located on Akyatan Beach within the provincial borders of Adana, Türkiye (Figure 1) as part of TÜBİTAK Scientific and Technological Research Projects Funding Program (project no: 122R096).

**Figure 2:**
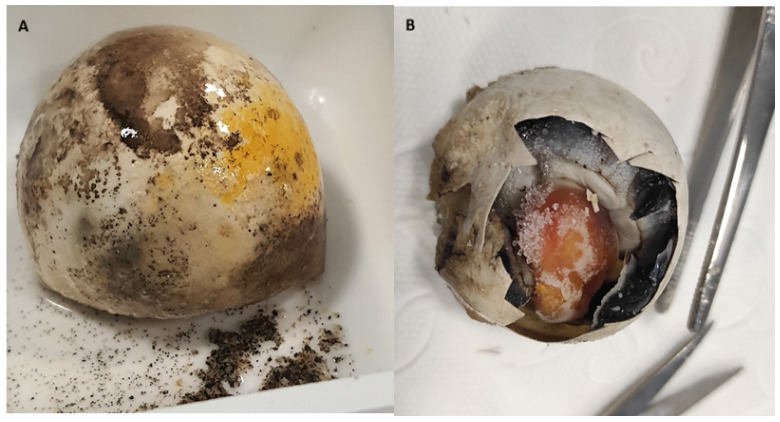
Egg samples. (**A**) Egg with an infection; (**B**) Dead embryo.

During the late August to early September period, which corresponds to the hatchling emergence season, a total of 30 distinct nests were sampled. Symptomatic eggs, dead embryos, and associated sand samples were aseptically collected from each nest (Figure 2). Sand samples were collected as a contingency measure; had bacterial isolation from eggshell fragments and dead embryos proven unsuccessful, sand samples would have served as an alternative source for bacterial recovery. As sufficient isolates were obtained directly from egg material, sand samples were not processed further in this study. All samples were transported on dry ice to the Hakkari University Biodiversity Application and Research Center. Egg samples were stored at −20 °C until further processing.

### 2.2. Bacterial Isolation and Identification

Bacterial isolation was performed using a modified version of the fungal isolation protocol described by Sarmiento-Ramírez et al. [31]. To remove adhering sand and debris, the surface of each egg collected from the nests was rinsed with sterile physiologic saline water (0.9%). Subsequently, the eggshells were aseptically cut into approximately 1 cm^2^ fragments using sterile scissors and placed at equal intervals onto Tryptic Soy Agar (TSA), Eosin Methylene Blue (EMB) agar, Xylose Lysine Deoxycholate (XLD) agar, and Salmonella-Shigella (SS) agar plates. All culture media used in this study were obtained from Merck (Darmstadt, Germany). The inoculated media were incubated at 37 °C for 48 h. This temperature was chosen because it represents the standard incubation temperature for the isolation of clinically relevant bacterial species of importance to both animal and human health, consistent with the One Health framework of this study. While we acknowledge that this temperature exceeds typical sea turtle nest incubation temperatures and may have reduced the recovery of bacteria better adapted to lower environmental temperatures, our primary aim was the isolation and characterization of bacteria with potential clinical significance rather than a comprehensive survey of nest-associated microbiota. Future studies combining multiple incubation temperatures would provide a more complete picture of bacterial diversity across the full thermal range of nesting environments. Subculturing was carried out under the same incubation conditions and on the same media types until pure cultures were obtained. Isolated pure bacterial cultures were transferred into 20% skim milk medium and stored at −20 °C for long-term preservation. Gram staining was performed to determine Gram reaction characteristics and cell morphology.

MALDI-TOF MS is fundamentally based on the ionization of proteins—particularly ribosomal proteins—present in biological samples, followed by the measurement of their mass-to-charge (*m*/*z*) ratios. The resulting mass spectrum serves as a unique “fingerprint” for each microorganism, enabling their accurate identification. The cultured isolates were inoculated onto selective media and subsequently sent to the Plant Health Clinic Application and Research Center at Hatay Mustafa Kemal University for MALDI-TOF MS analysis. Bacterial identification was performed using a Bruker Daltonik MALDI Biotyper system.

Bacterial isolates identified via MALDI-TOF MS analysis as potentially pathogenic—based on the relevant literature—were selected for molecular characterization. Genomic DNA was extracted using the GeneAll^®^ Exgene™ Cell SV Mini Kit (GeneAll Biotechnology, Seoul, Republic of Korea) according to the manufacturer’s instructions. DNA concentrations were measured using a Bioer QuantFlex 800 fluorometer (Bioer Technology, Hangzhou, China). This device utilizes fluorescent dye-based quantification, providing highly sensitive and accurate measurements of nucleic acid concentrations compared to conventional spectrophotometric methods.

Following DNA extraction, the 16S rRNA gene region was amplified by PCR using universal primers 27F (5′-AGA GTT TGA TCM TGG CTC AG-3′) and 1492R (5′-CGG TTA CCT TGT TAC GAC TT-3′) as described by Jiang et al. [32]. Polymerase chain reactions (PCR) were performed in a final volume of 25 µL using the Ampliqon 2× PCR Master Mix (Ampliqon, Odense, Denmark). Each reaction contained 12.5 µL of 2× PCR Master Mix, 1 µL of forward primer (10 µM), 1 µL of reverse primer (10 µM), 1 µL of template DNA (50 ng/µL), and nuclease-free water, resulting in a total volume of 25 µL.

The thermal cycling was conducted in a thermocycler (LongGene T20, Hangzhou, China) under the conditions given in Supplementary Table S1. PCR products were subsequently analyzed by agarose gel electrophoresis (Cleaver Scientific, Rugby, UK) with 2% agarose gel, at 90 V for 45 min and visualized under UV illumination (ER Biotech Gen-Box imagER CFx, Ankara, Türkiye). The PCR products were subsequently sent to Macrogen (Amsterdam, The Netherlands) for Sanger sequencing. The DNA sequences obtained from the company were compared with GenBank database entries using the BLASTn v2.17 tool available on the NCBI website, and species-level identification of the samples was subsequently performed.

### 2.3. Phylogenetic Analysis

A total of 11 16S rRNA gene sequences were retrieved and compiled for analysis. Multiple sequence alignment was performed using MAFFT v7.5 [33] with the L-INS-i strategy. *Vibrio vulnificus*, *Pseudomonas aeruginosa*, and *Burkholderia cepacia* were designated as outgroup taxa to root the phylogenetic tree. The best-fit nucleotide substitution model was determined using ModelFinder in IQ-TREE v3.0.1 [34,35]. Based on the Bayesian Information Criterion (BIC), the TN + F + R2 model was selected. Phylogenetic reconstruction was performed using the Maximum Likelihood (ML) method, and branch support was evaluated through 1000 ultrafast bootstrap (UFB) replicates. The tree was visualized using FigTree v1.4.4 [36].

### 2.4. Whole-Genome Sequencing and Bioinformatic Analysis

Whole-genome sequencing (WGS) was performed through a commercial service provided by Macrogen (The Netherlands). Genomic DNA from selected bacterial isolates was submitted to the company, where sequencing was conducted using the Illumina Nova SeqX platform.

#### 2.4.1. Library Preparation and Whole Genome Sequencing

Sequencing libraries were prepared using the Illumina TruSeq DNA PCR free (350) library preparation workflow according to the manufacturer’s instructions. The libraries were quantified with Qubit florometer. Paired-end sequencing (2 × 150 bp) was carried out on the Nova SeqX platform (Illumina Inc., San Diego, CA, USA), yielding an average genome coverage of 2 Gb × per sample.

#### 2.4.2. Quality Control and Read Processing

Raw sequence data were subjected to quality control using Trimmomatic v0.39 [37] software to remove low-quality reads, adapter sequences, and potential contaminants. High-quality reads were further evaluated using FASTQC v0.25.1 [38] for base quality, GC content, and overall sequencing metrics. Reads with a Q20 and Q30 score below 95% or shorter than 100 bp were discarded. Following filtering, the retained reads showed Q20 and Q30 values exceeding 99.8% and 97.8% for *C. farmeri*, and 98.8% and 95.4% for *E. cloacae*, respectively.

#### 2.4.3. Genome Assembly and Annotation

Filtered reads were assembled de novo using the Unicycler v0.5.1 [39], and assembly statistics such as N50, total length, and GC content were computed with tools like GC Content v1.0.2. The assembled contigs were mapped using CGView v2.1.0 [40]. *Citrobacter farmeri* strain FDAARGOS (GCA019048063.1) and *Enterobacter cloacae* isolate 1382 (GCF905331262.2) were used as reference genomes. Annotation of assembled genomes was performed with Bakta v1.9.4 [41]. The presence of tRNA and rRNA genes was predicted with Barrnap and Aragorn, respectively.

#### 2.4.4. Functional and Comparative Genomic Analyses

Functional annotation of coding sequences (CDSs) was carried out using the KEGG Pathway database, and pathway mapping was performed via the KEGG Automatic Annotation Server and PATRIC [42,43,44]. Antimicrobial resistance genes were identified with CARD Resistance Gene Identifier v1.3.0 [45], and virulence-associated genes were screened using the ABRicate v1.0.1 [46]. CRISPR loci were identified using CRISPRCasFinder v4.2.20 [47].

Comparative genomic analysis between isolates and reference genomes was conducted using FastANI v1.34 [48] to calculate average nucleotide identity (ANI). To explore shared and divergent gene content between the two isolates, ortholog clustering was performed using a custom Python v3.14.4 script implemented with Biopython v1.87 and NumPy v2.4 [49,50]. Given that this analysis involves only two genomes from different genera, the resulting core and accessory gene partitions should be interpreted as a preliminary comparative genomic overview rather than a conventional pangenome analysis. Pan-genome visualization was generated using Matplotlib v3.10.0 and Seaborn v0.13 [51,52].

### 2.5. Data Availability

The raw sequencing reads and assembled genomes were deposited in the NCBI GenBank under BioProject accession number PRJNA1399118. Genome annotations are accessible under GenBank accession numbers JBTMWS000000000 (*C. farmeri*) and JBTMWT000000000 (*E. cloacae*).

## 3. Results

### 3.1. Isolation and Morphological Characterization

From the 30 egg samples processed, a total of 15 discrete bacterial isolates were recovered. Primary selection was based on distinct colony phenotypes observed across a diverse range of selective and differential media (TSA, EMB, XLD, and SS). Microscopic evaluation via Gram staining showed a predominance of Gram-negative bacteria, with 75% exhibiting rod morphology.

### 3.2. Identification via MALDI-TOF MS

MALDI-TOF MS analysis provided robust species-level identification for the majority of the collection. Out of the 15 isolates analyzed, 12 were confidently identified with scores above 2.0, while isolate A2 (*Enterobacter cloacae*) received a score of 1.83, corresponding to a possible identification that was subsequently confirmed by 16S rRNA sequencing (Table 1). The final identified collection spanned 6 genera and 7 species. The most prevalent taxa were identified as Alcaligenes faecalis, while 2 of the strains were recognized as opportunistic or primary pathogens: *Citrobacter farmeri* and *Enterobacter cloacae*.

### 3.3. Molecular Validation and Phylogenetic Analysis

To corroborate MALDI-TOF MS findings, genomic DNA was extracted from 8 representative isolates. PCR amplification of the 16S rRNA gene (primers 27F/1492R) consistently produced high-intensity amplicons at the expected 1500 bp mark.

The subsequent BLASTn analysis of the sequences against the GenBank database revealed high identity values, ranging from 93–98%. While these values are modest for definitive species-level resolution—a known limitation of 16S rRNA-based identification within Enterobacteriaceae—they were sufficient for preliminary taxonomic placement. Species-level identification was ultimately confirmed through ANI analysis as described below. MALDI-TOF and 16S rRNA sequencing showed similar results for 8 isolates (Table 2).

**Figure 3:**
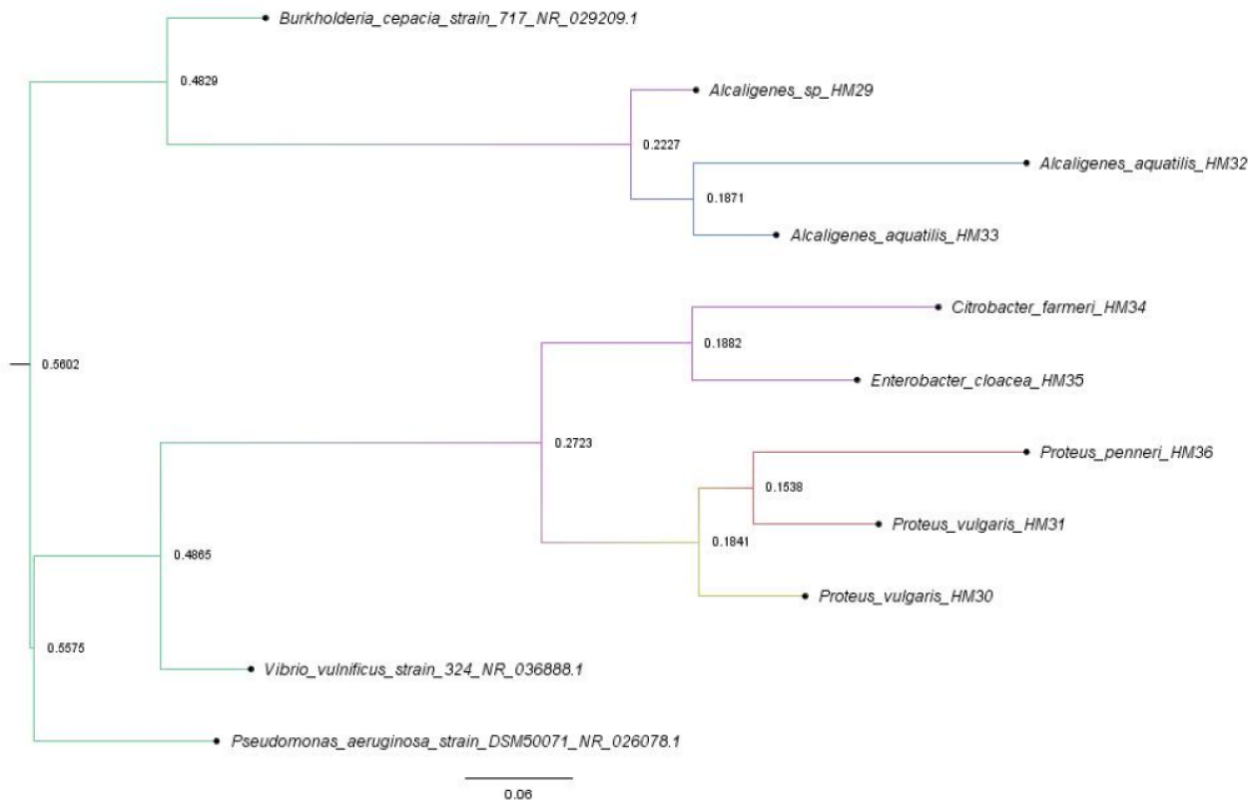
Maximum Likelihood (ML) phylogenetic tree based on 16S rRNA gene sequences of 11 isolates and reference strains. The tree was reconstructed using IQ-TREE with the TN + F + R2 substitution model. Numbers at the nodes represent bootstrap support values (%) based on 1000 ultrafast bootstrap replicates. Outgroup taxa (*Vibrio vulnificus*, *Pseudomonas aeruginosa*, and *Burkholderia cepacia*) were used to root the tree. The scale bar represents the number of substitutions per site.

The multiple sequence alignment comprised 11 sequences with 1742 nucleotide sites. Model selection identified the TN + F + R2 model as the best-fit substitution model for the dataset (BIC score: 20,828.45). The Maximum Likelihood (ML) tree revealed well-supported clades for the major groups. Notably, the enteric bacteria *Citrobacter farmeri* HM34 and *Enterobacter cloacae* HM35 formed a monophyletic group with high statistical confidence (Bootstrap Support [BS] = 100%) Figure 3. Similarly, *Alcaligenes aquatilis* and *Alcaligenes faecalis* HM29 clustered with strong nodal support (BS = 92%). The outgroup taxa provided a consistent rooting for the analysis, effectively separating the study groups from the distal lineages.

### 3.4. Distribution of Pathogenic Taxa

Based on both MALDI-TOF and 16S rDNA analysis results, two taxa known for their pathogenic potential in both chelonians and humans—*C. farmeri* and *E. cloacae*—were selected for further analysis.These results highlight a diverse and clinically relevant bacterial consortium associated with sea turtle eggshells, characterized by a mix of ubiquitous environmental microbes and specialized pathogens.

### 3.5. Whole-Genome Sequencing Output and Quality Assessment

Whole-genome sequencing of the selected bacterial isolates generated high-quality paired-end reads using the Illumina NovaSeq X platform. A total of 14,459,904 and 13,763,996 reads were obtained for *C. farmeri* and *E. cloacae*, corresponding to an average sequencing output of approximately 14,111,950 Gb per genome. After quality filtering and trimming, for *C. farmeri* 13,744,078 raw reads and for *E. cloacae* 12,914,526 raw reads were retained for downstream analyses.

**Figure 4:**
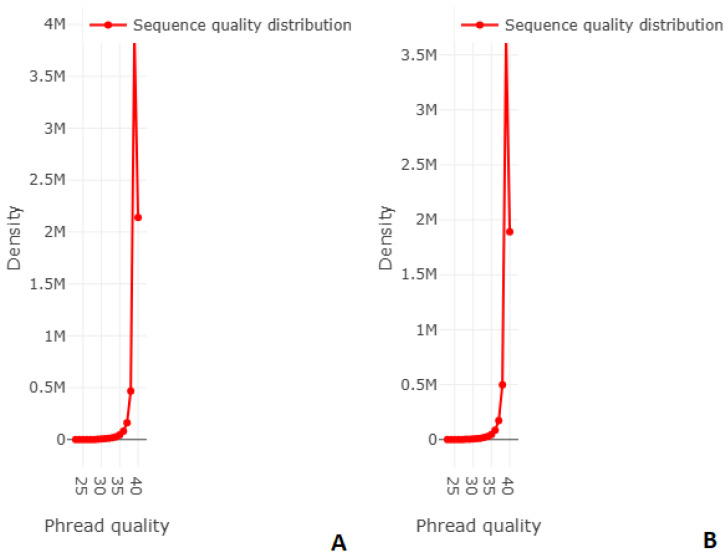
Sequence quality distribution of reads: (**A**) *C. farmeri* quality distribution; (**B**) *E. cloacae* quality distribution.

Quality assessment indicated consistently high base-call accuracy across all samples. The proportion of bases with Q20 and Q30 scores exceeded 99.8% and 97.8% for *C. farmeri* and 98.8% and 95.4% for *E. cloacae*, respectively (Figure 4). GC content distribution was uniform and consistent with values expected for members of the Enterobacteriaceae. GC contents are 53.2% for *C. farmeri* and 54.7% for *E. cloacae*.

### 3.6. Genome Assembly and Structural Characteristics

De novo assembly using the Unicycler pipeline yielded high-quality draft genomes for both the *Citrobacter* and *Enterobacter* isolates. The *Citrobacter* genome was resolved into 53 contigs with a total size of 5.1 Mb, while the *Enterobacter* assembly exhibited a slightly larger footprint of 5.2 Mb across 73 contigs. High assembly continuity was evidenced by N50 values of 321,096 bp and 256,670 bp for each isolate, respectively, ensuring reliable downstream genomic analyses. Notably, the GC content remained consistent across both genera at approximately 54%, aligning with the established taxonomic profiles for these Enterobacteriaceae members. A comprehensive breakdown of assembly metrics, including L50 and specific contig distributions, is provided in Table 3.

### 3.7. Genome Annotation

**Figure 5:**
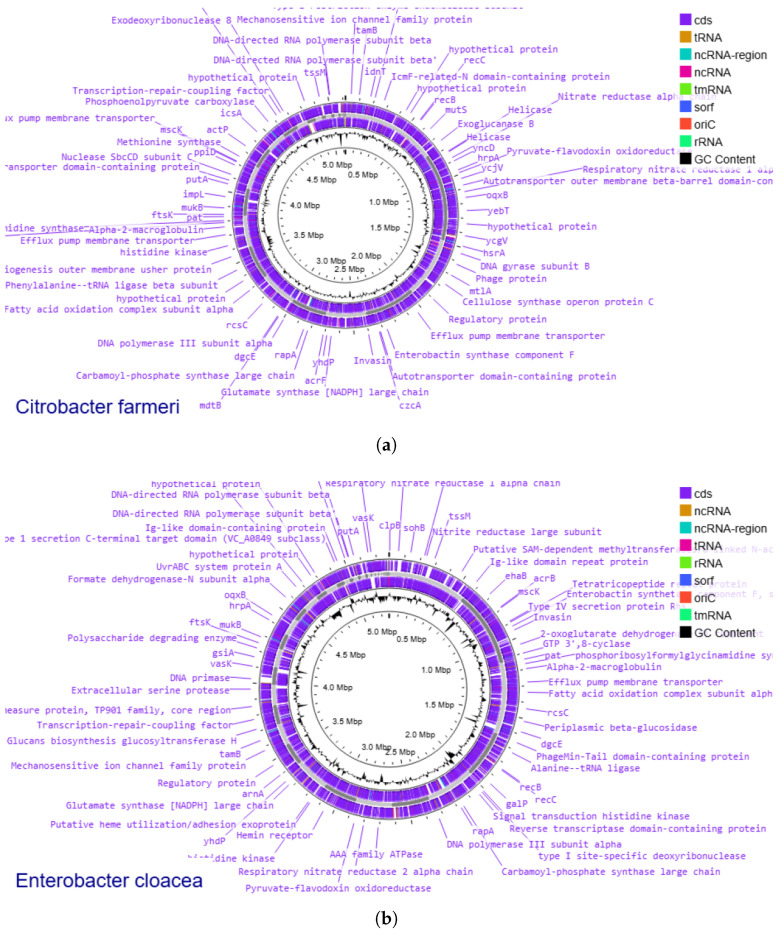
Whole genome maps of isolates: (**a**) *C. farmeri*; (**b**) *E. cloacae*.

Following the genome annotation, 4905 and 5071 protein-coding sequences (CDS) were identified for *C. farmeri* and *E. cloacae*, respectively (Figure 5). The number of tRNA genes and rRNA operons were determined to be 74 and 8 for *C. farmeri*, while *E. cloacae* exhibited 70 tRNA genes and 10 rRNA operons. In both cases, the predicted gene sets exhibit high similarity with standard housekeeping genes and regulatory proteins observed in closely related reference strains.

### 3.8. Functional Annotation and Metabolic Pathways

**Figure 6:**
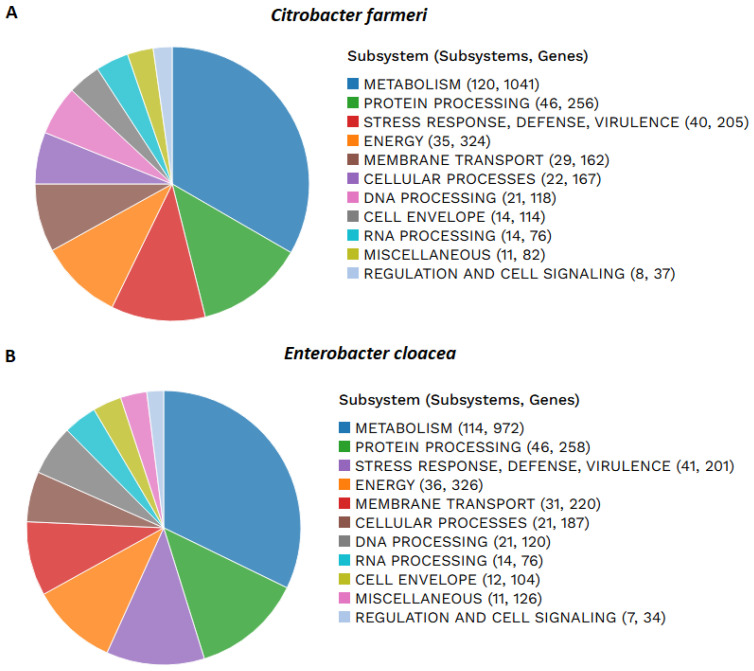
Functional distribution of genes in both isolates: (**A**) *C. farmeri*; (**B**) *E. cloacae*.

The functional distribution of the CDSs in both genomes revealed that cellular activities are predominantly devoted to core metabolic processes, including carbohydrate, amino acid, and energy production (Figure 6). Furthermore, a group of genes representing 6.85% of the sequences in *C. farmeri* and 7.56% in *E. cloacae* were classified as hypothetical proteins with functions that remain to be characterized.

Both genomes exhibited a robust metabolic framework, particularly dominated by categories such as Metabolic Pathways, Biosynthesis of secondary metabolites, and Microbial metabolism in diverse environments. Metabolic reconstruction further unveiled complete or near-complete biosynthetic modules for Carbon and Nitrogen metabolism, underscoring the physiological versatility of these isolates within their respective niches. While the general pathway distribution remained largely congruent between the two strains, subtle variations in Lipid and Xenobiotics metabolism, and pathways related to diseases suggest niche-specific adaptations. These functional distributions of each pathway group are given in Supplementary Tables S2 and S3.

### 3.9. Antimicrobial Resistance, Virulence-Associated Genes and CRISPR-Cas Systems

Comprehensive screening against the Comprehensive Antibiotic Resistance Database (CARD) revealed that the *Citrobacter* and *Enterobacter* isolates carry 38 and 37 predicted antimicrobial resistance (AMR) gene sequences, respectively (Supplementary Figure S1). The predicted resistome profiles were predominantly characterized by gene sequences associated with fluoroquinolone, aminoglycoside, and β-lactam resistance. Notably, the identification of gyrA, gyrB, SugE and AmpC gene sequences suggests a genomic landscape consistent with both intrinsic and potentially acquired resistance mechanisms. While the predicted AMR gene counts were relatively similar, the Enterobacter isolate showed a higher proportion of fluoroquinolone-associated sequences, suggesting a potentially more specialized predicted resistance repertoire. These findings represent genotypic predictions and require phenotypic validation through antimicrobial susceptibility testing to confirm functional resistance.

Parallel screening for virulence-associated gene sequences via ABRicate identified 71 and 52 sequences, respectively, spanning categories such as adhesion, iron sequestration, and stress response. The presence of these gene sequences indicates a genomic repertoire consistent with opportunistic pathogenicity, though functional assays would be required to confirm their expression and activity in the nest environment.

CRISPR-CasFinder analysis detected 4 CRISPR loci across the analyzed genomes, 2 in *C. farmeri* and 2 in *E. cloacae*. No CAS loci were detected in any genomes.

### 3.10. Comparative Genomics and ANI Analysis

**Figure 7:**
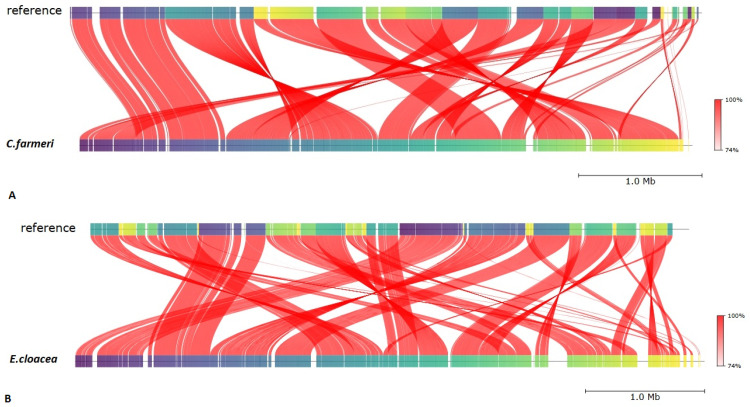
ANI comparisons of isolates. The colored blocks on the top and bottom bars represent conserved genomic regions (syntenic blocks). The red ribbons connecting the two genomes indicate nucleotide identity levels, as shown in the color scale. Crossed ribbons highlight structural rearrangements, such as inversions, while parallel ribbons indicate maintained strand orientation: (**A**) *C. farmeri*; (**B**) *E. cloacae*.

To resolve the taxonomic boundaries of the isolates, we performed Average Nucleotide Identity (ANI) analysis, which confirmed a robust genomic affinity between our strains and their respective reference counterparts (Figure 7).

The ANI values for the two isolates were calculated as 99.13% for *Citrobacter farmeri* and 98.48% for *Enterobacter cloacae*, both well above the 95–96% species delimitation threshold. Detailed pairwise ANI comparisons between these isolates and related type strains are documented in Table 4.

Beyond species identification, comparative genomic profiling unveiled a distinct architecture characterized by highly conserved core regions interspersed with variable accessory elements. This mosaic gene distribution highlights a significant degree of genomic plasticity, likely facilitating the environmental adaptation and niche specialization observed within these *Citrobacter* and *Enterobacter* lineages.

### 3.11. Comparative Genomic Analysis of Shared and Divergent Gene Content

**Figure 8:**
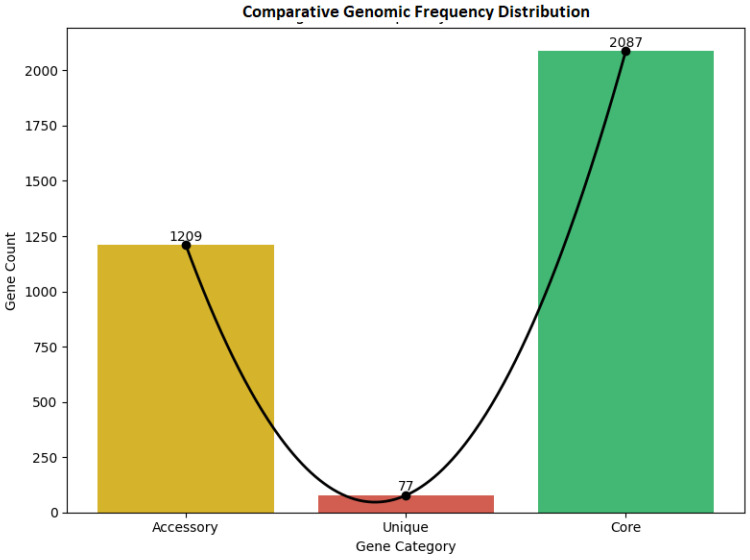
Comparative genomic frequency distribution of orthologous gene clusters identified between *Enterobacter cloacae* and *Citrobacter farmeri*. The black solid line represents the fitted U-shaped frequency distribution curve.

Comparative genomic analysis of the two isolates identified a total of 3373 orthologous gene clusters. Among these, 2087 clusters were shared by both isolates, representing approximately 61.87% of the total gene repertoire and likely encompassing broadly conserved housekeeping functions. The remaining clusters were partitioned into 1209 isolate-specific accessory genes and 77 unique genes. Given that this comparison involves only two genomes from different genera, these figures reflect intergeneric genomic differences rather than intraspecies pangenomic variation, and should be interpreted accordingly (Figure 8).

This partitioning underscore the genomic flexibility of the *Citrobacter* and *Enterobacter* isolates, where the accessory and unique components likely drive niche-specific adaptations and specialized metabolic traits. The architectural distribution of these shared and divergent orthologous groups is visualized in comparative genomic frequency distribution plot in Figure 8, highlighting the balance between evolutionary conservation and genomic innovation. Comparative genomic heatmap of first 50 genes were given in Supplementary Figure S2.

## 4. Discussion

Our study involved a detailed phenotypic and genomic characterization of two bacterial isolates recovered from unhatched sea turtle eggs, *Citrobacter farmeri* and *Enterobacter cloacae*. By combining MALDI-TOF MS, 16S rRNA, and whole-genome sequencing (WGS), we were able to validate these isolates with high taxonomic precision. While these results are based on two sequenced genomes and should be interpreted as preliminary, they provide useful insight into the genomic traits of these bacteria within the nesting beach context.

The high prevalence of Gram-negative, rod-shaped bacteria observed in our samples aligns with earlier findings on reptilian nests and coastal sands [31,53,54,55,56,57,58]. In such environments, Enterobacteriaceae family, *Pseudomonas*, and the *Alcaligenes* genera typically thrive, likely due to their metabolic flexibility and ability to withstand harsh environmental conditions. By identifying 15 isolates across six different genera with MALDI-TOF, we provide a preliminary indication of the bacterial diversity present on eggshell surfaces in this nesting site. This heterogeneity is consistent with turtle nests functioning as complex microhabitats influenced by both terrestrial and marine environments, though broader sampling would be needed to characterize this diversity more fully. Of particular interest is the frequent recovery of *Alcaligenes*; its ability to survive in saline, nutrient-poor settings supports the idea that this species may be a core component of the nest microbiota rather than a transient occupant.

In our analysis, MALDI-TOF MS proved to be a highly effective screening tool; 12 out of 15 strains yielded identity scores above 2.0, allowing for rapid and reliable preliminary identification. This proteomic approach was further supported by the high degree of congruence we observed with 16S rRNA sequencing across eight representative isolates. However, the 16S rRNA sequences showed relatively modest identity values (93–98%), reflecting a common challenge in Enterobacteriaceae systematics [59,60,61]. In this group, conserved ribosomal loci and frequent horizontal gene transfer often limit the resolution of single-gene phylogenetics. To overcome this, we employed ANI analysis, which provided the necessary taxonomic clarity [62]. Since both isolates surpassed the 95–96% species boundary, we were able to definitively assign them to *C. farmeri* and *E. cloacae*.

Our Maximum Likelihood phylogenetic tree further confirmed these taxonomic assignments, placing our isolates in close evolutionary proximity to both clinical and environmental reference strains. This overlap suggests that the lineages of *Citrobacter* and *Enterobacter* are not rigidly divided into ‘pathogenic’ or ‘environmental’ categories. Instead, they likely exist along a continuum, driven by genomic plasticity and niche-specific selection. The mosaic-like architecture we observed in both genomes—where highly conserved core regions are interspersed with variable accessory elements—is a hallmark of the Enterobacteriaceae family. We argue that this genomic flexibility is exactly what enables these bacteria to adapt to such diverse and challenging ecological contexts, including the unique interface of marine and terrestrial environments found in turtle nests.

From a genomic perspective, the assembly metrics for both isolates reflect high-quality draft genomes, providing a reliable basis for our comparative and functional analyses. With genome sizes of approximately 5.1–5.2 Mb and GC contents around 54%, these strains align closely with the established genomic profiles of *Citrobacter* and *Enterobacter* [63,64,65]. The N50 values, all exceeding 250 kb, ensure strong structural continuity, which allowed us to confidently annotate coding sequences, rRNA operons, and tRNA genes. Furthermore, the gene counts and functional repertoires we identified mirror those of reference genomes [66]. This suggests that while these isolates maintain canonical housekeeping and regulatory systems, they also possess specific accessory functions that likely enhance their fitness in the challenging environment of a nesting beach.

Functional annotation highlighted a metabolic profile centered on carbohydrate, amino acid, and energy metabolism. This broad catabolic versatility likely enables these bacteria to persist on nutrient-variable substrates, such as eggshells and the surrounding sand. The discovery of near-complete carbon and nitrogen metabolic modules further suggests that both strains are well-equipped to exploit diverse organic resources [67,68,69]—whether derived from nest materials, embryonic exudates, or the broader microbial community. Interestingly, we noted subtle differences between the strains regarding lipid and xenobiotic metabolism. These variations may point toward micro-niche specialization or perhaps a history of exposure to anthropogenic contaminants, a possibility that warrants more focused investigation in future studies.

The predicted AMR gene repertoire detected in both genomes is noteworthy. The identification of 38 and 37 AMR-associated gene sequences in *C. farmeri* and *E. cloacae* respectively—spanning fluoroquinolones, aminoglycosides, and β-lactams—reveals a predicted resistome profile that is consistent with those reported in clinical and environmental Enterobacteriaceae [70,71,72]. The co-occurrence of gyrA/gyrB and AmpC gene sequences is consistent with a combination of intrinsic and potentially acquired resistance mechanisms, though phenotypic susceptibility testing would be needed to confirm functional resistance. The higher proportion of fluoroquinolone-associated sequences in *E. cloacae* may suggest exposure to selective pressures such as agricultural runoff or anthropogenic pollutants [73,74,75], a pattern observed in coastal and estuarine microbiomes, though this remains speculative without direct environmental data.

The routes by which AMR genes may have reached this conservation nesting environment warrant deeper consideration. Akyatan Beach, despite its protected status, is situated within a region subject to multiple anthropogenic pressures. Agricultural activity and urban development in the surrounding coastal zone contribute to diffuse pollution through surface runoff and wastewater discharge, which are well-documented vectors for the introduction of antibiotic residues and resistance determinants into coastal sediments and nearshore waters [73,74,75]. The selective pressure exerted by sub-inhibitory concentrations of antibiotics in such environments can promote the maintenance and horizontal transfer of AMR genes within microbial communities, potentially explaining the fluoroquinolone and β-lactam resistance profiles observed in our isolates.

However, anthropogenic runoff may not be the sole pathway. Nesting female sea turtles, which spend the majority of their lives in the open ocean and coastal foraging grounds, may serve as carriers of environmental bacteria acquired during their migratory routes. Given that marine environments are increasingly recognized as reservoirs of AMR determinants—particularly in areas affected by aquaculture, shipping, and coastal urbanization—it is plausible that females could introduce bacteria with acquired resistance genes directly to the nest during oviposition. Vertical transmission of bacteria from females to eggs has been previously documented in sea turtles [20,21], and this pathway deserves further investigation as a potential route of AMR introduction in nesting environments.

Disentangling these pathways—whether AMR genes arrived via environmental contamination of the beach, through the nesting females, or through a combination of both—will require longitudinal studies combining microbial source tracking, environmental sampling, and analysis of female cloacal microbiota alongside nest microbiota. Such an integrated approach would provide a clearer picture of how AMR is introduced and maintained in sea turtle nesting ecosystems within a One Health framework.

The detection of virulence-associated gene sequences—specifically those related to adhesion, iron acquisition, and stress response—indicates that these isolates possess genomic features commonly associated with opportunistic pathogenicity in Enterobacteriaceae. While the presence of these gene sequences does not confirm active virulence or host infection, their abundance suggests that these strains carry the genomic potential for opportunistic behavior under permissive conditions [76,77,78,79]. Functional assays and experimental infection models would be necessary to determine whether these genes are expressed and contribute to eggshell colonization or embryonic infection. Whether these traits are expressed in the nest environment and whether they contribute to egg failure remains to be determined through functional and experimental studies [21,56,80].

Interestingly, our CRISPR-Cas analysis identified multiple CRISPR loci in both genomes, yet no detectable Cas genes were found. This configuration likely reflects relic or incomplete adaptive immune systems [81]. Such truncated architectures are not uncommon in Enterobacteriaceae and typically point to historical phage exposure rather than active functional immunity [82]. From an evolutionary perspective, the lack of Cas proteins could make these strains more permissive to horizontal gene transfer [83]. This ‘genomic openness’ would facilitate the acquisition of accessory genes related to resistance, metabolism, or virulence, potentially explaining the remarkable plasticity and adaptive breadth we observed in these isolates.

Comparative genomic analysis identified 3373 orthologous gene clusters between the two isolates. The 2087 shared clusters likely represent broadly conserved functions common to Enterobacteriaceae [84,85,86,87,88], while the accessory and unique gene pools may reflect genus-level or isolate-specific traits. However, given that this comparison involves only two genomes from different genera, we caution against interpreting these figures in the conventional pangenome sense. Expanding this analysis to include multiple strains within each species would be necessary to draw meaningful conclusions about intraspecies genomic plasticity or openness.

The isolate-specific gene clusters may reflect genus-level differences in metabolic capacity and environmental responsiveness, including potential differences in stress tolerance and substrate utilization [84,89,90]. The presence of unique genes in each isolate is consistent with the genomic flexibility known in Enterobacteriaceae, though functional characterization and broader sampling would be needed to confirm whether these genes represent recent horizontal gene transfer events or longstanding genus-specific traits, such as shifting nutrient levels or intense microbial competition. This balance between conserved and variable orthologous groups demonstrates a dynamic interplay between evolutionary stability and the innovation required to thrive in fluctuating coastal environments.

The distribution of core versus accessory genes has significant consequences for both the pathogenic potential and the environmental resilience of these isolates. While the core genome maintains fundamental physiological functions, the accessory and unique gene pools likely act as ‘genetic toolkits’—providing the traits needed for host association, antimicrobial resistance, and ecological competitiveness. Within a One Health framework, this genomic flexibility allows these bacteria to cross the boundaries between environment, wildlife, and humans. This reinforces the idea that nesting beaches may serve as active exchange zones for adaptive genetic elements. From a conservation standpoint, this comparative genomic structure suggests that microbial communities in sea turtle nests are not static; instead, they are evolutionarily responsive systems capable of rapid functional shifts as they encounter anthropogenic and environmental pressures.

## 5. Conclusions

By using phenotypic, molecular, and genomic data, we demonstrate that sea turtle eggshells can host bacteria with clinically significant genomic traits, including predicted multidrug-resistant and virulence-associated gene content. These results broaden our current understanding of nest-associated microbiota, showing that bacterial opportunists—not just fungi—warrant further investigation as potential contributors to egg failure, though a causal role cannot be established from genomic data alone. From a conservation standpoint, the detection of these taxa in unhatched eggs suggests a need for further investigation into the bacterial communities of nesting beaches. While our findings are preliminary given the small number of sequenced isolates, they support the potential value of incorporating microbial monitoring into nesting beach management protocols, especially in areas heavily impacted by human activity and tourism.

Beyond its technical and genomic findings, our study has significant implications for sea turtle conservation, particularly within the ‘One Health’ framework—an approach that highlights the deep link between environmental, animal, and human health. Finding opportunistic Enterobacteriaceae with predicted multidrug-resistant and virulence-associated genomic profiles on eggshells points to a potentially overlooked area of investigation in nesting ecosystem health. While conservation efforts regarding egg mortality have historically focused on fungal pathogens, our data suggest that bacterial agents with clinically relevant genomic traits warrant further investigation as potential contributors to embryonic health outcomes, pending phenotypic and functional validation.

From a conservation perspective, finding *C. farmeri* and *E. cloacae* in nesting substrates suggests that microbial pollution—driven by wastewater, anthropogenic runoff, or tourism—may be fundamentally altering the microbial ecology of these sites. We are concerned that such shifts could select for more resilient and pathogenic bacterial communities, adding another layer of risk to already vulnerable sea turtle populations. These findings suggest that traditional physical and ecological monitoring of beaches is no longer enough; we must also incorporate routine microbial surveillance into management protocols. In practice, this would mean periodic screening of sand and eggshell microbiota, using genomic profiling to catch emerging resistance or virulence trends before they lead to significant reproductive losses.

The ‘One Health’ implications of our findings are underscored by the clear genomic proximity between our isolates and clinical reference strains. That environmental, wildlife, and human-pathogenic lineages occupy the same phylogenetic space highlights how easily ecological boundaries can be crossed. This suggests a bidirectional flow of microbes and resistance genes across seemingly separate ecosystems. Nesting beaches, which sit at the delicate interface of land and sea, are frequently exposed to human activity and may act as silent reservoirs or ‘exchange hubs’ for antimicrobial resistance. This raises a critical possibility: wildlife-associated bacteria may be contributing to the broader environmental resistome. Such a trend could have serious consequences for public health, especially in coastal communities that depend on nearshore waters for recreation, fishing, or their daily livelihoods.

Ultimately, our findings suggest that wildlife conservation should no longer be viewed strictly as a biodiversity issue, but as an essential component of public health. The genomic signatures of resistance and virulence we detected in turtle-associated bacteria show exactly how human-driven pressures can ripple through microbial ecosystems and reappear at the interface of wildlife and human activity. Consequently, future studies should expand genomic sampling across multiple isolates and species, enabling proper pangenomic analyses that can more robustly characterize the microbial risks associated with sea turtle nesting environments. This approach would allow for the creation of early warning systems to detect pathogenic threats in sensitive nesting habitats, enabling evidence-based interventions that protect both endangered species and the broader health of coastal environments.

While our findings offer significant insights, several limitations must be considered. The small number of isolates used for WGS, for instance, means that our genomic inferences should be viewed as a preliminary baseline rather than a broad generalization. Expanding this sampling across different nesting seasons and more diverse geographic locations will be essential to map out long-term temporal and spatial trends. Additionally, we did not perform phenotypic antimicrobial susceptibility testing; therefore, the resistance determinants identified in the genomes remain genotypic predictions that require phenotypic validation. Finally, although we identified numerous virulence factors, functional assays are still needed to confirm their actual expression and to clarify the complex interactions between these bacteria and the host embryo.

In summary, our study provides a detailed look at the genomic and functional traits of *C. farmeri* and *E. cloacae* found on sea turtle eggshells, uncovering a significant overlap between environmental resilience, antibiotic resistance, and virulence. These findings reveal the hidden ecological complexity of nest-associated microbiomes and demonstrate why genomic surveillance is a vital tool for modern conservation research. To fully understand the epidemiological impact of these bacteria, future work should focus on longitudinal sampling and experimental infection models. Such efforts will be key to developing evidence-based strategies to protect endangered marine turtles from emerging microbial threats.

## Figures and Tables

**Table 1:** MALDI-TOF MS results of the isolates.

Sample	Organism	Identity Score
A1	*Citrobacter farmeri*	2.26
A2	*Enterobacter cloacae*	1.83
A3	Not reliable identification	1.34
A4	*Pseudomonas aeruginosa*	2.15
A5	*Alcaligenes faecalis*	2.26
A6	*Alcaligenes faecalis*	2.25
A7	*Acinetobacter pittii*	2.15
A8	*Alcaligenes faecalis*	2.24
A9	*Proteus vulgaris*	2.26
A10	*Pseudomonas aeruginosa*	2.42
A11	*Bacillus cereus*	2.12
A12	*Pseudomonas aeruginosa*	2.36
A13	Not reliable identification	1.33
A14	*Proteus vulgaris*	2.40
A15	*Pseudomonas aeruginosa*	2.35

Identity score: 2.3–3.0 high identification; 2.0–2.29 normal identification; 1.7–1.99 possible identification; 1.69 and below not reliable identification.

**Table 2:** Molecular identification results with 16S rDNA region.

Sample	Species	Accession No
A1	*Citrobacter farmeri* HM34	PX999052
A2	*Enterobacter cloacae* HM35	PX999053
A3	*Alcaligenes faecalis* HM29	PX999051
A6	*Alcaligenes aquatilis* HM32	PX999049
A8	*Alcaligenes faecalis* HM33	PX999050
A9	*Proteus vulgaris* HM30	PX999055
A14	*Proteus cibi* HM36	PX999054
A15	*Proteus vulgaris* HM31	PX999056

**Table 3:** Assembly metrics of the isolates.

	*C. farmeri*	*E. cloacae*
Genome Size (bp)	5,118,851	5,282,041
Number of Contigs	53	73
N50 (bp)	321,096	256,670
L50	5	7
GC	53.2%	54.7%

**Table 4:** ANI Results.

	*C. farmeri*	*E. cloacae*
Avarage Nucleotide Identity (ANI)	99.13%	98.48%
Reference Strain	*Citrobacter farmeri* strain FDAARGOS	*Enterobacter cloacae* isolate 1382
Query Sequence Fragments	1689	1673
Orthologous Matches	1535	1500

## Data Availability

The DNA sequences obtained in this study were deposited in GenBank, and their accession numbers are provided in the manuscript.

## Supplementary Materials

The following supporting information can be downloaded at: https://www.mdpi.com/article/10.3390/vetsci13050462/s1, Figure S1: Map of antimicrobial resistance genes; Figure S2: Comparative Genomic heatmap of first 50 genes of isolates and their reference genomes; Table S1: PCR conditions; Table S2: Metabolic Pathways of *Citrobacter farmeri*; Table S3: Metabolic Pathways of *Enterobacter cloacea*.
