# Supplementary material for: Whole-Genome Sequencing and Comparative Genomic Analysis of Citrobacter farmeri and Enterobacter cloacae from Unhatched Green Turtle Eggs
Source: Vet Sci. 2026 May 10;13(5):462. doi: 10.3390/vetsci13050462 (PMC13211643; doi:10.3390/vetsci13050462)
Supplement: Supplementary file 1 [file vetsci-13-00462-s001.zip › vetsci-4281502-supplementary.pdf]

Whole Genome Sequencing and Pan-genomic Analysis of *Citrobacter farmeri* and *Enterobacter cloacea* from Green Turtle (*Chelonia mydas*) Eggs

Nurcan ÖNEN[1], Bahadır TÖRÜN[2]*, Can YILMAZ[2]

Supplementary Material

Supplementary Table 1. PCR conditions

	**Temp (°C)**	**Time**	**Cycle**
**Initial Denaturation**	95	5 minutes	
**Denaturation**	95	30 seconds	35
**Annealing**	58	30 seconds	
**Extension**	72	30 seconds	
**Final Extension**	72	5 minutes	

Supplementary Table 2. Metabolic Pathways of *Citrobacter farmeri*

		Pathway	KEGG ID	Pathways Found	Enzymes
Metabolism	Global	Metabolic Pathways	01100	7	formate dehydrogenase, glutamate synthase, acetate kinase, type I pantothenate kinase, phosphomannomutase, adenylate cyclase, acyl-CoA dehydrogenase
		Biosynthesis of secondary metabolites	01110	3	glutamate synthase, 5-methyltetrahydrofolate--homocysteine methyltransferase, phosphomannomutase
		Microbial metabolism in diverse environments	01120	2	glutamate synthase, formate dehydrogenase
		Carbon metabolism	01200	1	formate dehydrogenase
		Fatty acid metabolism	01212	1	acyl-CoA dehydrogenase
		Biosynthesis of amino acids	01230	2	5-methyltetrahydrofolate--homocysteine methyltransferase, glutamate synthase
		Biosynthesis of nucleotide sugars	01250	1	phosphomannomutase
		Biosynthesis of cofactors	01240	1	type I pantothenate kinase
	Carbohydrate metabolism	Fructose and mannose metabolism	00051	1	phosphomannomutase
		Glyoxylate and dicarboxylate metabolism	00630	1	formate dehydrogenase
	Energy metabolism	Methane metabolism	00680	1	formate dehydrogenase
		Nitrogen metabolism	00910	1	glutamate synthase (NADPH)
	Lipid metabolism	Fatty acid degradation	00071	1	acyl-CoA dehydrogenase
	Nucleotide metabolism	Purine metabolism	00230	1	adenylate cyclase, class 1

	Amino acid metabolism	Alanine, aspartate and glutamate metabolism	00250	1	glutamate synthase (NADPH)
		Cysteine and methionine metabolism	00270	1	5-methyltetrahydrofolate--homocysteine methyltransferase
	Metabolism of other amino acids	Selenocompound metabolism	00450	1	5-methyltetrahydrofolate--homocysteine methyltransferase
	Glycan biosynthesis and metabolism	Amino sugar and nucleotide sugar metabolism	00520	1	phosphomannomutase
	Metabolism of cofactors and vitamins	Pantothenate and CoA biosynthesis	00770	1	type I pantothenate kinase
		One carbon pool by folate	00670	1	5-methyltetrahydrofolate--homocysteine methyltransferase
Genetic Information Processing	Transcription	RNA polymerase	03020	1	DNA-directed RNA polymerase subunit beta
	Replication and repair	DNA replication	03030	1	DNA polymerase III subunit alpha
		Nucleotide excision repair	03420	1	transcription-repair coupling factor (superfamily II helicase)
		Mismatch repair	03430	1	DNA polymerase III subunit alpha
		Homologous recombination	03440	1	DNA polymerase III subunit alpha
Cellular Processes	Cellular community - prokaryotes	Quorum sensing	02024	1	bapA; large repetitive protein
		Biofilm formation	05111, 02026	3	adenylate cyclase, class 1, diguanylate cyclase
Organismal Systems	Digestive system	Folate transport and metabolism	04981	1	5-methyltetrahydrofolate--homocysteine methyltransferase
		Cobalamin transport and metabolism	04980	1	5-methyltetrahydrofolate--homocysteine methyltransferase
	Environmental adaptation	Plant-pathogen interaction	04626	1	elongation factor Tu
Human Diseases	Drug resistance: antineoplastic	Antifolate resistance	01523	1	5-methyltetrahydrofolate--homocysteine methyltransferase

Supplementary Table 3. Metabolic Pathways of *Enterobacter cloacea*

		**Pathway**	**KEGG ID**	**Pathways Found**	**Enzymes**

Metabolism	Global	Metabolic Pathways	01100	10	formate dehydrogenase major subunit, nitrite oxidoreductase, alpha subunit, chitinase, urea/guanidine carboxylase, quinate dehydrogenase, penicillin-binding protein 1A, adenylate cyclase class 1, proline dehydrogenase, (R)-2-hydroxyglutarate dehydrogenase
Metabolism	Global	Biosynthesis of secondary metabolites	01110	4	5-methyltetrahydrofolate--homocysteine methyltransferase, urea/guanidine carboxylase, quinate dehydrogenase (quinone), delta 1-pyrroline-5-carboxylate dehydrogenase
Metabolism	Global	Microbial metabolism in diverse environments	01120	3	formate dehydrogenase major subunit, (R)-2-hydroxyglutarate dehydrogenase, nitrate reductase
Metabolism	Global	Carbon metabolism	01200	1	formate dehydrogenase major subunit
Metabolism	Global	Biosynthesis of amino acids	01230	1	5-methyltetrahydrofolate--homocysteine methyltransferase
Metabolism	Global	Nitrogen cycle	01310	1	nitrate reductase / nitrite oxidoreductase, alpha subunit Transporters
Metabolism	Carbohydrate metabolism	Glyoxylate and dicarboxylate metabolism	00630	1	formate dehydrogenase major subunit
Metabolism	Energy metabolism	Methane metabolism	00680	1	formate dehydrogenase major subunit
Metabolism	Energy metabolism	Nitrogen metabolism	00910	1	nitrite oxidoreductase, alpha subunit
Metabolism	Nucleotide metabolism	Purine metabolism	00230	1	adenylate cyclase, class 1
Metabolism	Amino acid metabolism	Alanine, aspartate and glutamate metabolism	00250	1	delta 1-pyrroline-5-carboxylate dehydrogenase
Metabolism	Amino acid metabolism	Cysteine and methionine metabolism	00270	1	5-methyltetrahydrofolate--homocysteine methyltransferase
Metabolism	Amino acid metabolism	Lysine degradation	00310	1	(R)-2-hydroxyglutarate dehydrogenase
Metabolism	Amino acid metabolism	Arginine biosynthesis	00220	1	urea/guanidine carboxylase

		Arginine and proline metabolism	00330	1	delta 1-pyrroline-5-carboxylate dehydrogenase
		Phenylalanine, tyrosine and tryptophan biosynthesis	00400	1	quinate dehydrogenase (quinone)
	Metabolism of other amino acids	Selenocompound metabolism	00450	1	5-methyltetrahydrofolate--homocysteine methyltransferase
	Glycan biosynthesis and metabolism	Amino sugar and nucleotide sugar metabolism	00520	1	chitinase
		Peptidoglycan biosynthesis	00550	1	penicillin-binding protein 1A
	Metabolism of cofactors and vitamins	One carbon pool by folate	00670	1	5-methyltetrahydrofolate--homocysteine methyltransferase
	Xenobiotics biodegradation and metabolism	Atrazine degradation	00791	1	urea/guanidine carboxylase
Genetic Information Processing	Transcription	RNA polymerase	03020	1	DNA-directed RNA polymerase subunit beta
	Replication and repair	DNA replication	03030	1	single-strand DNA-binding protein
		Nucleotide excision repair	03420	1	transcription-repair coupling factor (superfamily II helicase)
		Mismatch repair	03430	1	single-strand DNA-binding protein
		Homologous recombination	03440	2	single-strand DNA-binding protein, exodeoxyribonuclease V beta subunit
Environmental Information Processing	Membrane transport	ABC transporters	02010	1	homopolymeric O-antigen transport system ATP-binding protein
		Bacterial secretion system	03070	1	type VI secretion system protein ImpL
	Signal transduction	Two-component system	02020	1	nitrite oxidoreductase, alpha subunit
Cellular Processes	Cellular community - prokaryotes	Quorum sensing	02024	1	large repetitive protein
		Biofilm formation	05111, 02025, 02026	3	adenylate cyclase class 1, type VI secretion system protein ImpL
Organismal Systems	Digestive system	Folate transport and metabolism	04981	1	5-methyltetrahydrofolate--homocysteine methyltransferase

		Cobalamin transport and metabolism	04980	1	5-methyltetrahydrofolate--homocysteine methyltransferase
	Environmental adaptation	Plant-pathogen interaction	04626	1	elongation factor Tu
Human Diseases	Drug resistance: antineoplastic	Antifolate resistance	01523	1	5-methyltetrahydrofolate--homocysteine methyltransferase
	Infectious disease: bacerial	Pertussis	05133	1	filamentous hemagglutinin
	Drug resistance: antimicrobial	beta-Lactam resistance	01501	2	penicillin-binding protein 1A, multidrug efflux pump
		Cationic antimicrobial peptide (CAMP) resistance	01503	1	multidrug efflux pump

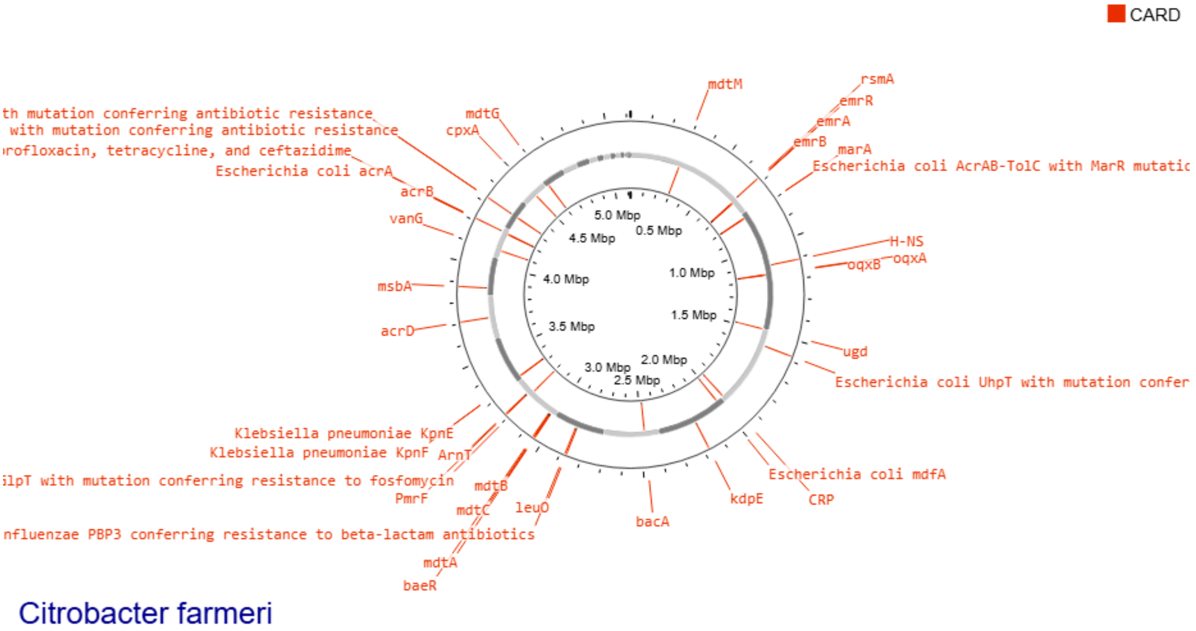

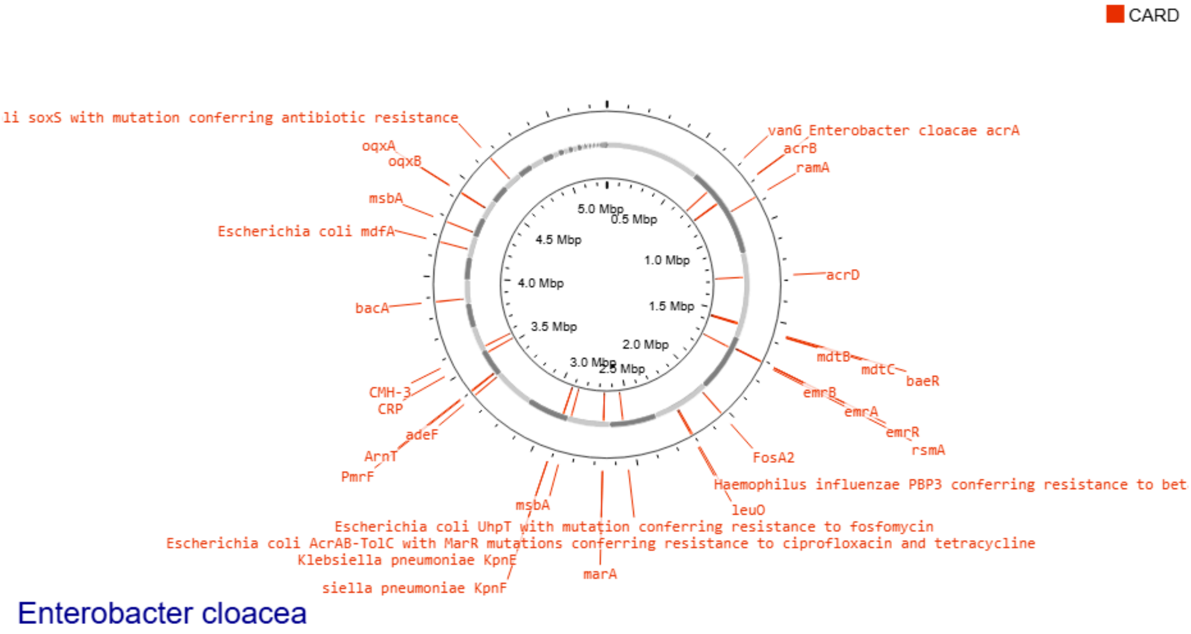

Supplemantary Figure 1.Map of antimicrobial resistance genes.

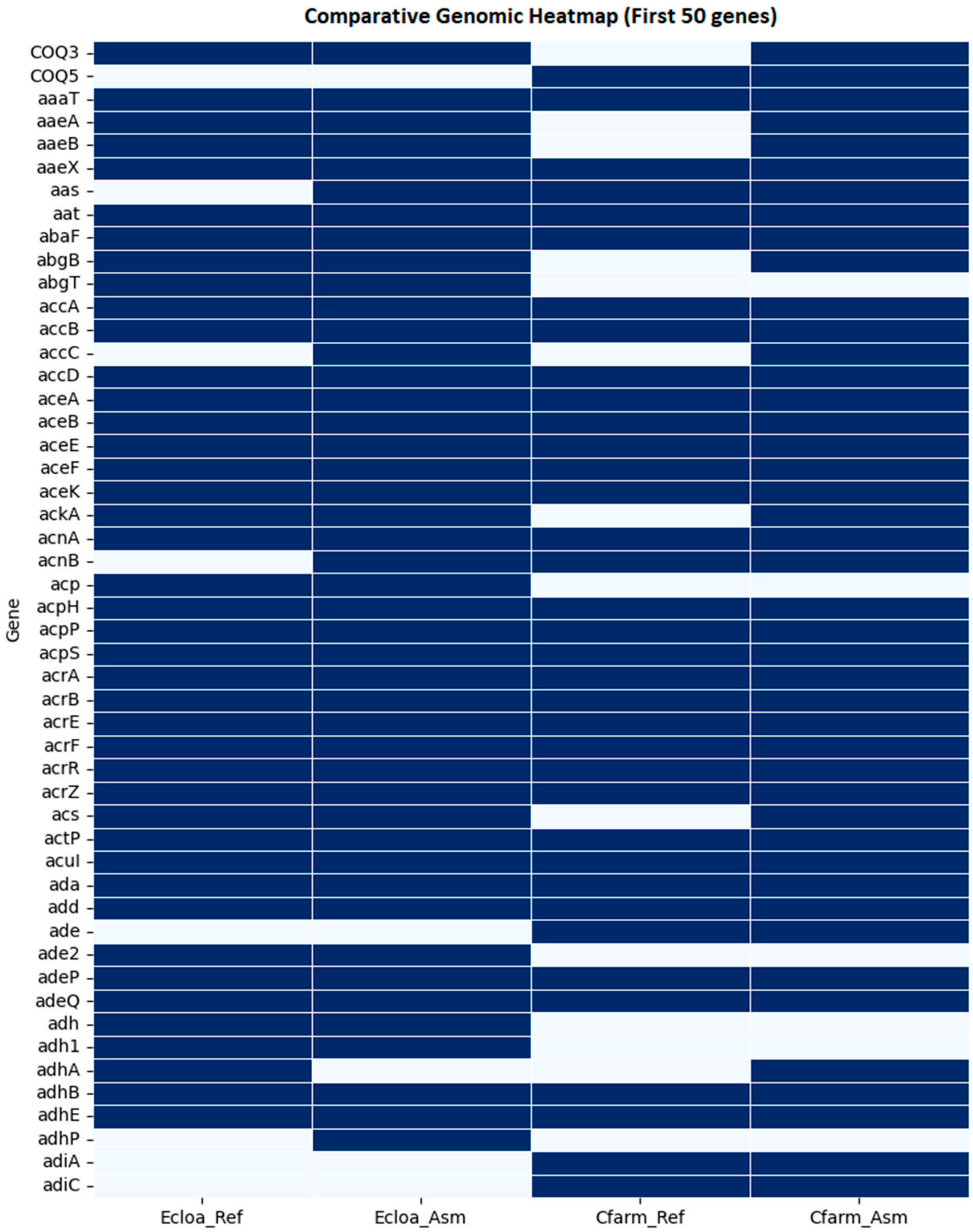

Supplementary Figure 2. Comparative Genomic heatmap of first 50 genes of isolates and their reference genomes.
